# Machine learning approach of automatic identification and counting of blood cells

**DOI:** 10.1049/htl.2018.5098

**Published:** 2019-07-17

**Authors:** Mohammad Mahmudul Alam, Mohammad Tariqul Islam

**Affiliations:** Department of EEE, Bangladesh University of Engineering and Technology, Dhaka, Bangladesh

**Keywords:** learning (artificial intelligence), patient diagnosis, blood, object detection, image classification, cellular biophysics, medical image processing, neural nets, complete blood cell count, machine learning approach, automatic identification, counting, blood smear images, red blood cells, white blood cells, blood cells detection

## Abstract

A complete blood cell count is an important test in medical diagnosis to evaluate overall health condition. Traditionally blood cells are counted manually using haemocytometer along with other laboratory equipment's and chemical compounds, which is a time-consuming and tedious task. In this work, the authors present a machine learning approach for automatic identification and counting of three types of blood cells using ‘you only look once’ (YOLO) object detection and classification algorithm. YOLO framework has been trained with a modified configuration BCCD Dataset of blood smear images to automatically identify and count red blood cells, white blood cells, and platelets. Moreover, this study with other convolutional neural network architectures considering architecture complexity, reported accuracy, and running time with this framework and compare the accuracy of the models for blood cells detection. They also tested the trained model on smear images from a different dataset and found that the learned models are generalised. Overall the computer-aided system of detection and counting enables us to count blood cells from smear images in less than a second, which is useful for practical applications.

## 1 Introduction

A complete blood cell (CBC) count is an important test often requested by medical professionals to evaluate health condition [1, 2]. The main three types of cells that constitute blood are red blood cells (RBCs), white blood cells (WBCs), and platelets. RBCs also known as erythrocytes are the most common type of blood cell, which consists of 40–45% of blood cells [American Society of Haematology: http://www.hematology.org/Patients/Basics/]. Platelets also known as thrombocytes are also in huge number in blood. WBCs also known as leukocytes, are just 1% of total blood cells. RBCs carry oxygen to our body tissues and the amount of oxygen tissues receives is affected by the number of RBCs. WBCs fight against infections and platelets help with blood clotting. As these blood cells are huge in number, traditional manual blood cell counting system using haemocytometer is highly time consuming and erroneous and most of the cases accuracy vastly depends on the skills of a clinical laboratory analyst [3, 4]. Therefore, an automated process to count different blood cells from a smear image will greatly facilitate the entire counting process.

With the development of machine learning techniques, image classification and object detection applications are becoming more robust and more accurate. As a result, machine learning based methods are being applied in different fields. Particularly, deep learning methods are being applied in different medical applications such as abnormality detection and localisation in chest X-rays [5], automatic segmentation of the left ventricle in cardiac MRI [6], and detection of diabetic retinopathy in retinal fundus photographs [7]. Thus, it is worth to look into deep learning based methods that can be applied to identify and count the blood cells in the smear images.

**Fig. 1:**
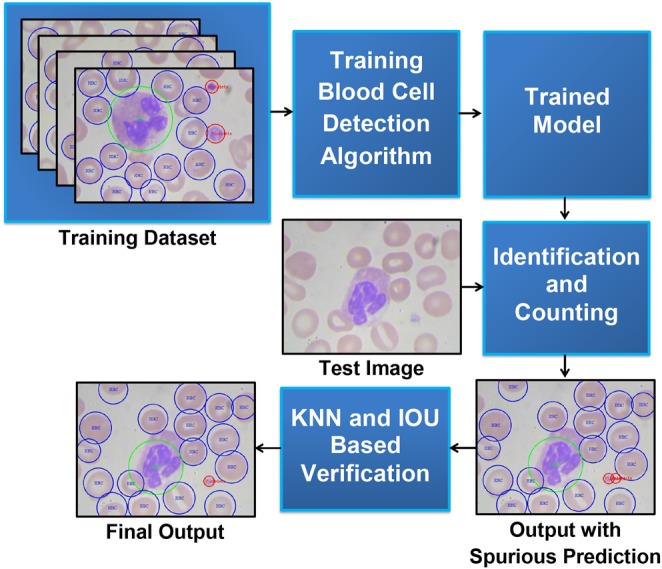
Block diagram of automatic blood cells identification and counting system

In this Letter, a deep learning based blood cell counting method has been proposed. We employ a deep learning based object detection method to detect different blood cells. Among the state-of-the-arts object detection algorithms such as regions with convolutional neural network (R-CNN) [8], you only look once (YOLO) [9], we chose YOLO framework which is about three times faster than Faster R-CNN with VGG-16 architecture [9]. YOLO uses a single neural network to predict bounding boxes and class probabilities directly from the full image in one evaluation. We retrain YOLO framework to automatically identify and count RBCs, WBCs, and platelets from blood smear images. To improve the counting accuracy, a verification method has been developed to avoid repeated counting by the framework. Also, the trained model has been tested with images from another dataset to observe the generalisation of the method. Fig. 1 shows the proposed deep learning based blood cell identification and counting system.

## 2 Related works

In general, there are generally two different approaches in the automated counting process of blood cells. They are the image processing approach [1, 3, 10–12] and the machine learning approach [2, 4, 13–15].

Acharya and Kumar [10] proposed an image processing technique for RBCs count. It processed the blood smear image to count RBCs along with the identification of normal and abnormal cells. They used the K-medoids algorithm to extract WBCs from the image and granulometric analysis to separate the RBCs from WBCs and then counted the number of cells using the labelling algorithm and a circular Hough transform (CHT). Sarrafzadeh *et al.* [11] proposed circlet transform to count RBCs on the greyscale image. They used iterative soft-thresholding method for identification and counting purposes. Kaur *et al.* [12] proposed a method to count platelets automatically by applying a CHT in a microscopic blood cells image. They used the size and shape features of platelets from the CHT in the counting process.

Cruz *et al.* [1] presented an image processing system to count blood cells. They used hue, saturation, value thresholding method, and connected component labelling for the identification and counting of blood cells. Acharjee *et al.* [3] proposed a semi-automated process by applying a Hough transform to count RBC by detecting their oval and biconcave shape. Lou *et al.* [4] provided a method to automatically count RBCs using spectral angle imaging and support vector machine (SVM). Zhao *et al.* [13] proposed an automatic identification and classification system for WBCs using the convolutional neural network (CNN). Firstly, they detected WBCs from the microscopic images, and then CNN was used to detect kinds of WBCs. Habibzadeh *et al.* [2] presented a system for classifying five different types of WBCs. They used three classifiers, which include two different SVMs and one CNN classifier. Habibzadeh *et al.* [14] employed pre-trained CNNs, ResNet [16] and Inception Net [17], to count WBCs from segmented images. The images were segmented employing colour space analysis. Xu *et al.* [15] employed patch size normalisation on pre-processed images and then applied CNN to classify RBC shapes from microscopy images of patients of sickle cell disease.

We propose a completely different approach that employs YOLO to detect all three types of blood cells simultaneously. Our method does not require any greyscale conversion or binary segmentation. The whole process is fully automated, fast, and accurate.

## 3 Materials and method

Our goal is to use the object detection and classification algorithm YOLO to detect and count blood cells directly from smear image. We need to train the YOLO framework with a modified configuration and annotated blood cells training images.

### 3.1 Dataset

We use a publicly available dataset of annotated blood cell images called Blood Cell Count Dataset (BCCD) [BCCD: https://github.com/Shenggan/BCCD_Dataset]. Originally it has a total of 364 annotated smear images, but the dataset has some crucial flaw. After splitting the dataset into training (300) and testing (64) parts, we find that one annotation file in the test set does not include any RBC, although the image contains RBCs. Moreover, three annotations file exhibit very low RBC than actual. So, we remove four fallacious files and the total size of the test set becomes 60. For the validation set, we randomly pick 60 training images with annotations. This modified dataset can be downloaded from this GitHub repository https://github.com/MahmudulAlam/Complete-Blood-Cell-Count-Dataset. To test our model on a different dataset, we used the data from [11]. The dataset includes 100 images of resolution $3246 \times 2448$ acquired by Nikon V1 camera mounted on a Nikon ECLIPSE 50i microscope with a magnification of 100×.

### 3.2 YOLO

‘You Only Look Once’ in short YOLO is a state-of-the-art object detection classification algorithm [9]. It treats object detection as a regression problem. It requires only one forward propagation pass through the network to make a fast prediction for both image class and location. It resizes the image by $448 \times 448$ and divides the entire image into a $7 \times 7$ grid cell and each grid cell predicts for two bounding boxes and confidence score for the boxes. If the centre of the object falls into a grid cell that the grid cell is responsible for detecting that object. The original implementation of the YOLO model as a CNN evaluated on the PASCAL VOC dataset. Its network architecture contains 24 convolutional layers and 2 fully connected layers and inspired by the GoogLeNet. Among different versions of it, we choose to use Tiny YOLO as it is the fastest of all. Tiny YOLO uses 9 instead of 24 convolutional layers other than that all the parameters are the same [9].

### 3.3 Training

The original implementation of the Tiny YOLO configuration was trained for 20 different classes. To adopt it for blood cells identification, we modify it for three classes consisting of WBC, RBC, and platelets. Due to modifying the class number, the number of filters in the final convolutional layer in the CNN architecture is needed to be changed as well. YOLO predicts five values along with class probabilities for each anchor box. The values are the probability of having an object in a grid cell, *x* and *y* coordinates of the object, height, and width of the object. In our case, the number of anchor boxes is 5 as it will provide better flexibility to put bounding boxes according to the aspect ratio of the object [18]. The number of filters in the final convolution layer, $N_F$, can be computed from the number of anchor boxes $N_A$ and number of classes $N_C$ by

(1) $$N_F = N_A \times \lpar N_C + 5\rpar .\eqno\lpar 1\rpar $$

Since $N_A$ is 5 and $N_C$ in 3 in the experiments, $N_F$ is found to be 40.

**Fig. 2:**
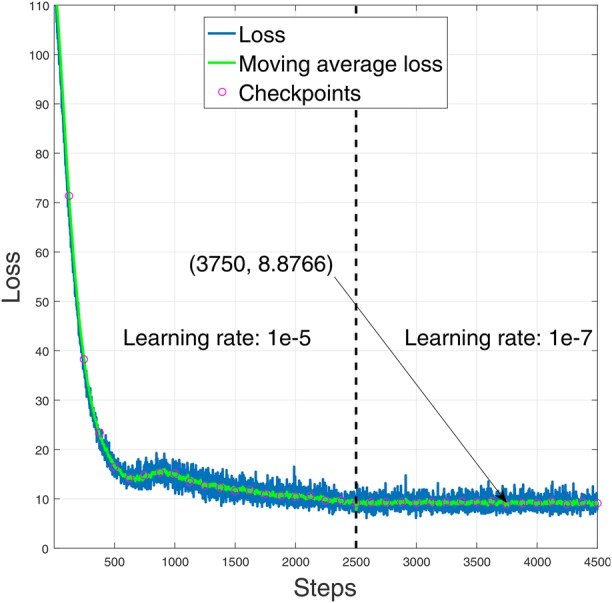
Learning curve of the YOLO framework for blood cell identification

We use 300 annotated blood smear images for training and 60 for testing. During training in each step, we record loss and moving average loss. We record data for a total of 4500 steps and use two different learning rates. For steps 1–2500, we specify the learning rate $10^{ - 5}$ and for steps 2501–4500, the learning rate $10^{ - 7}$. We found that, a lower learning rate at the later steps enables better convergence. We recorded the weights and evaluated the model after every 125 steps. Fig. 2 shows the learning curve of the YOLO algorithm for blood cell detection in terms of the loss function. The value of the loss function is shown as is as well as by the moving average of the loss function. It is seen from the figure that, the minimum moving average loss is found to be 8.8766 on step 3750 using the learning rate $10^{ - 7}$. We use the weights of this step for testing purpose.

### 3.4 Proposed blood cell identification and counting method

**Fig. 3:**
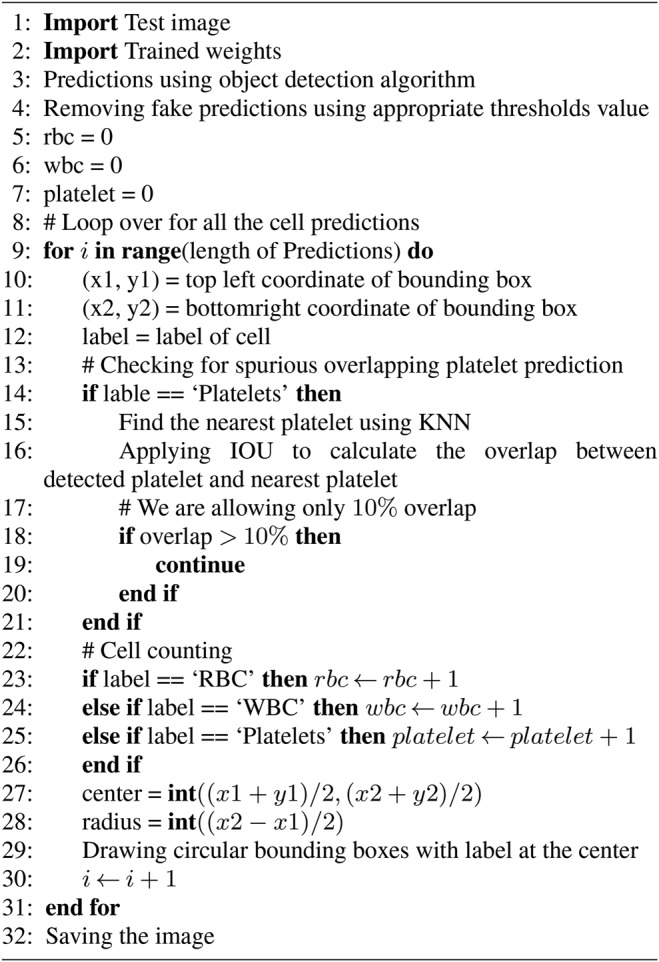
Algorithm 1: automatic blood cell identification and counting

Our proposed method is a machine learning approach where we use YOLO algorithm for automatic identification and counting of blood cells. It includes a training model with a modified configuration where we change the final convolution layer for three outputs, identification of blood cells with an appropriate threshold, and count them from their labels. We choose the threshold value by calculating the average absolute error between ground truths and our estimation at difference threshold value and realise the appropriate threshold for each type of cell that gives a minimum average absolute error in the validation dataset. Our proposed method does not misinterpret among cells such as identifying RBC as WBC or platelets as RBC and so on. In some cases, it double count platelets. We resolve this by using K-nearest neighbour (KNN) and intersection over union (IOU) in each platelet. Overall, our proposed method is fast and accurate in the identification and counting of blood cells. The steps of the proposed method are described in Algorithm 1 (see Fig. 3).

We can get four parameters from the YOLO model for each detected cell. They are the label of the cell, the confidence of being that cell, top left corner position, and bottom right corner position. To show which of the cells are detected in the blood smear image, we have two choices. Using the top left and bottom right corner coordinates, we can put a rectangular bounding box that encloses each detected cell. However, blood cells are not rectangular rather close to circular in shape, and rectangular boxes occupy much redundant space than it requires. So, we place circular bounding boxes to enclose each cell, and that requires the conversion of the top left and bottom right coordinates to radius and centre of the circle.

Given the top left and bottom right coordinates are $\lpar x_1\comma \; y_1\rpar $ and $\lpar x_2\comma \; y_2\rpar $, the centre point *C* and the radius *r* of the circle that encloses the cell can be calculated by

(2) $$C = \left({\displaystyle{{x_1 + x_2} \over 2}\comma \; \displaystyle{{y_1 + y_2} \over 2}} \right)\eqno\lpar 2\rpar $$

(3) $$r = \left({\displaystyle{{x_2 - x_1} \over 2}} \right)\eqno\lpar 3\rpar $$

We count cells using their label. The modified YOLO returns three kinds of labels ‘RBC’, ‘WBC’, and ‘Platelets’ depending on the detected cell. The total number of RBC in a smear image will be the total number of labels containing ‘RBC’, the total number of WBC will be the total number of labels containing ‘WBC’ and so on.

**Fig. 4:**
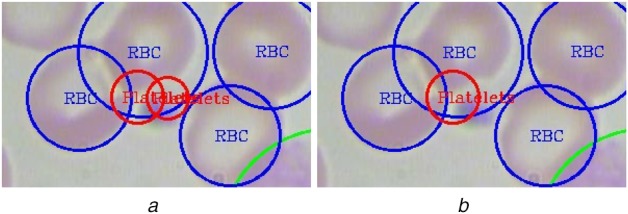
Example blood smear image showing *a* Counting of the same platelet twice *b* Discarding spurious prediction using the proposed method

In some cases, our models provide two different detections for a single platelet. We observed that the reason is the detection of the same platelet from two consecutive grid cells, and thus the same platelet is counted twice. To avoid this double counting problem, we apply the KNN algorithm in each platelet and determine its closest platelet and then using the intersection of union (IOU) between two platelets we calculate their extent of overlap. Using empirical observations, we allow 10% of the overlap between platelet and its closest platelet. If the overlap is larger than that, we ignore that cell as double count to get rid of spurious counting. Fig. 4 shows such a case where a platelet is detected twice by the YOLO algorithm. Using the proposed KNN and IOU based technique, this double detection problem has been removed.

## 4 Experiments and results

With the proposed method, we automatically identify and count RBCs, WBCs, and platelets. We test our model using a test dataset of 60 images where the ground truths are known. First, we use our model to count the different cells in the validation dataset with different confidence threshold. It is noted that the threshold plays an instrumental role in YOLO as it uses this threshold to predict each grid cell, not for the whole image. Grid cell containing no blood cell has low confidence. So, we can get rid of redundant and spurious predictions by choosing appropriate confidence threshold.

We calculate the average absolute error between ground truths and the estimated number of cells in the validation dataset. With different confidence threshold, we realise the minimum average absolute error value for each type of cell and choose those confidence values in the identification process of blood cells. The error is computed using

(4) $$\varepsilon ^{{\rm cell}} = \displaystyle{1 \over N}\sum\limits_{i = 1}^N \vert \chi _{{\rm groundtruths}}^{\lpar i\rpar } - \chi _{{\rm estimated}}^{\lpar i\rpar } \vert \eqno\lpar 4\rpar $$

where *cell* indicates the type of cells (RBC, WBC, or platelets), *N* is the size of validation dataset (in our experiment it is 60), $\chi $ is the number of cells, $\varepsilon $ is the average absolute error value for the particular cell. The computed error values are shown in Table 1. It is seen from the table that for counting RBCs, we can employ a nominal threshold of 0.55. However, for WBC and platelets, the threshold is found to be much lower (0.35 and 0.25 in our experiments). Thus, the appropriate thresholds for each type of cell are selected as follows:

- RBC: confidence threshold of 55%.
- WBC: confidence threshold of 35%.
- Platelets: confidence threshold of 25%.

**Table 1:** Average absolute error ground truths and estimated number of RBCs, WBCs, and platelets at a different confidence threshold

Threshold, %	RBC	WBC	Platelets
20	5.650	0.083	0.217
25	4.417	0.050	**0.083**
30	3.450	0.033	0.083
35	2.750	**0.017**	0.083
40	2.500	0.050	0.083
45	2.183	0.100	0.100
50	2.133	0.150	0.100
55	**2.083**	0.200	0.117
60	2.100	0.333	0.150

Then, we have calculated the accuracy from the total number of ground truths cells and the total number of estimated cells in the test dataset. With a confidence threshold of 55% for RBC, we achieved 96.09% accuracy for RBC. Total estimated numbers of cells of different types with accuracy calculated at their appropriate confidence threshold value are presented in Table 2. It may seem that the proposed algorithm is counting more or extra RBCs that are not in the images. However, we would like to note that the ground truth labels were not present for some of the RBCs that are at the edge of the image. The YOLO algorithm can detect these RBCs, and thus, the RBC count is high.

**Table 2:** Accuracy of counting RBC, WBC, and platelets employing the proposed method

	RBCs	WBCs	Platelets
ground truths	792	61	55
estimated	823	53	53
accuracy, %	96.09	86.89	96.36

**Fig. 5:**
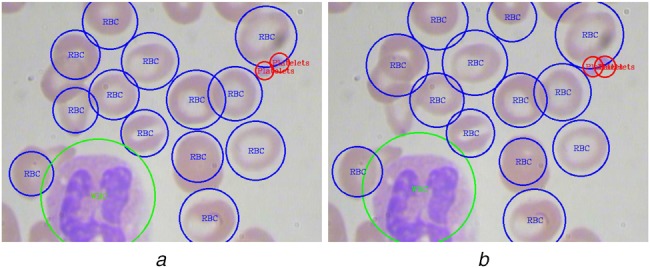
Comparison of the ground truth and predicted blood cell identification output *a* Ground truth labels of cells in a smear image *b* Automatically estimated labels of cells by our model

To visualise, the output of the proposed method concerning ground truth, a sample smear image from the test set is shown in Fig. 5. It is seen from the figure that all the WBC and platelets are detected without error. The method has missed one RBC in the middle, whereas detected another RBC from the edge of the image which is not present in the ground truth.

### 4.1 Experiments with other CNN architectures

**Fig. 6:**
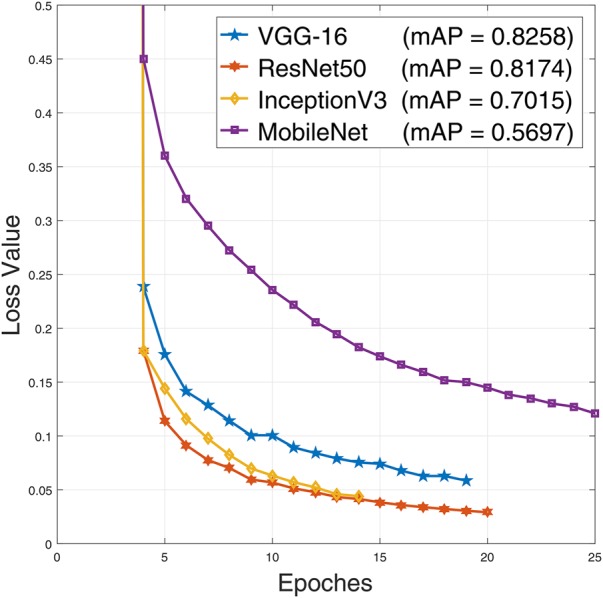
CNN models loss curves with YOLO algorithm along with their validation mAP

YOLO has a built-in CNN architecture for classification which is inspired by GoogLeNet architecture. Besides training blood cell detection model with YOLO's own CNN, we have experimented with other popular CNN architectures. We use VGG-16 [19], ResNet50 [16], InceptionV3 [17], and MobileNet [20] CNN architectures with the YOLO algorithm by replacing it's built-in CNN. To train YOLO using these networks in the backend, we have split our training dataset into two parts. First 250 images with annotations are used for training purposes and rest 50 s are used for validation purposes. For all the networks training loss curves along with validation mean average precision (mAP) values are shown in Fig. 6. It is seen from the figure that, InceptionV3 and ResNet50 achieves the lowest error.

The accuracy of counting RBC, WBC, and platelets on the test set is shown in Table 3. It is seen from the table that, the highest accuracy for counting RBCs and platelets is found for the Tiny YOLO architecture, which achieved 96.09% accuracy in counting RBCs and 96.36% accuracy in counting platelets. On the other hand, VGG-16 and InceptionV3 both have achieved the highest accuracy in counting WBCs (100%). Thus, different models can achieve the highest accuracy for different cells. We have also computed the mean average precision (mAP) of these CNN architectures and their execution time per test image listed in Table 3. It is seen from the table that ResNet50 achieves highest mAP value. Our proposed method is extremely fast requiring less than a second even for deeper networks. We calculate the forward propagation time for each of the test images on a computer with Intel Core i5 6500 with 8 GB memory and Nvidia GTX1050 Ti GPU with 4 GB memory. Estimation time is reported by computing the average to get the final execution time.

**Table 3:** Accuracy of detecting different cells using different CNN architecture with YOLO algorithm

		RBC	WBC	Platelet	mAP	Execution time, ms
	Ground truths	792	61	55		
Tiny YOLO	estimated	823	53	53	0.6236	60
	accuracy, %	**96.09**	86.89	**96.36**		
VGG-16	estimated	1006	61	60	0.7132	106
	accuracy, %	72.98	**100**	90.91		
ResNet50	estimated	952	58	48	0.7437	118
	accuracy, %	79.80	95.08	87.27		
InceptionV3	estimated	889	61	57	0.6826	130
	accuracy	87.75	**100**	**96.36**		
MobileNet	estimated	588	57	46	0.5207	84
	accuracy, %	74.24	93.44	83.64		

### 4.2 Testing using a different dataset

**Fig. 7:**
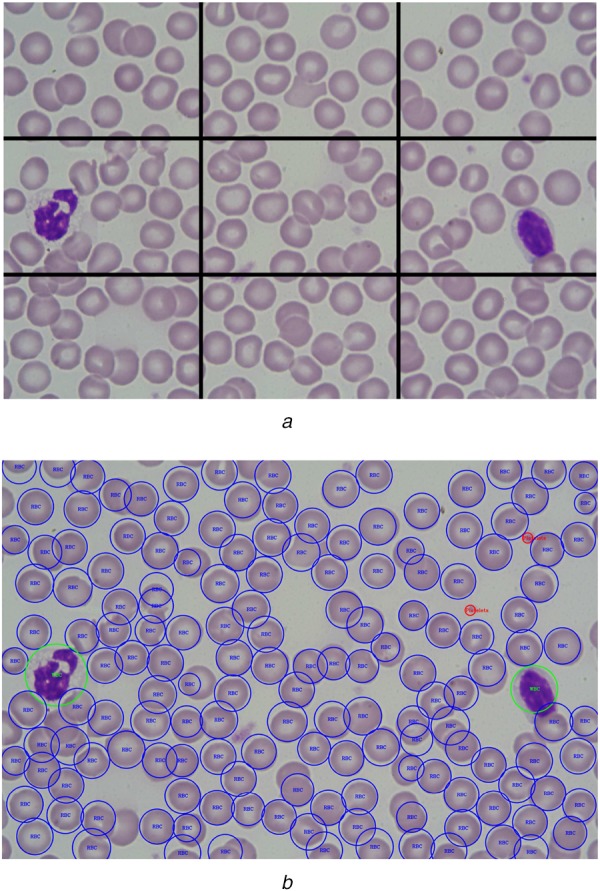
Blood cells detection in an image from the dataset [11] *a* Image divided into $3 \times 3$ grids *b* Combined output

To observe, whether the trained model is database dependent, we have also tested our model using blood smear images from another dataset [11]. The images of this dataset were of higher resolution, and thus the images were divided into grids to create sub-images, and each of the sub-images has been processed individually in the proposed pipeline. The detection and counting from each of the sub-images are then projected back on the original image. A blood smear image which has been divided into a $3 \times 3$ grid, which causes 9 sub-images to be processed in the proposed method, is shown in Fig. 7. It is seen from the figure that, the output image correctly identifies the RBCs, WBCs, and platelets with satisfactory performance.

## 5 Conclusion

In this Letter, a machine learning approach to automatically identify and count blood cells from a smear image based on YOLO algorithm is presented. To improve accuracy, the method employed KNN and IOU based method to remove multiple counting of the same object. Our proposed method is evaluated on publicly available datasets. It is observed for test dataset that, our method accurately identifies RBCs, WBCs, and Platelets. It is seen that our method can accurately count even some of the cells that are not labelled in the dataset. Different neural network models have also been tried in the YOLO back-end, and it has been observed that different models can provide the best accuracy on different cells. Even though different models with different depths have been tried, it is observed that the method is considerably fast for counting and marking the smear images. The proposed method has also been tested on a different dataset of smear images, where it has performed satisfactorily. With the accuracy and the detection performance of the proposed method, it can be said that, the method has the potential to ease up the manual blood cell identification and counting process.
